# Targeting PCSK9 in Liver Cancer Cells Triggers Metabolic Exhaustion and Cell Death by Ferroptosis

**DOI:** 10.3390/cells12010062

**Published:** 2022-12-23

**Authors:** Malak Alannan, Hala Fatrouni, Véronique Trézéguet, Franziska Dittrich-Domergue, Patrick Moreau, Géraldine Siegfried, Benjamin Liet, Abdel-Majid Khatib, Christophe F. Grosset, Bassam Badran, Hussein Fayyad-Kazan, Aksam J. Merched

**Affiliations:** 1 Bordeaux Institute of Oncology (BRIC), Inserm U1312, University of Bordeaux, 33000 Bordeaux, France; 2 Laboratoire de Biogenèse Membranaire, CNRS, UMR 5200, University of Bordeaux, 33140 Villenave d’Ornon, France; 3 Laboratory of Cancer Biology and Molecular Immunology, Faculty of Sciences I, Lebanese University, Beirut 90656, Lebanon

**Keywords:** PCSK9, ferroptosis, hepatocellular carcinoma, hepatoblastoma, lipid metabolism

## Abstract

Deregulated lipid metabolism is a common feature of liver cancers needed to sustain tumor cell growth and survival. We aim at taking advantage of this vulnerability and rewiring the oncogenic metabolic hub by targeting the key metabolic player pro-protein convertase subtilisin/kexin type 9 (PCSK9). We assessed the effect of PCSK9 inhibition using the three hepatoma cell lines Huh6, Huh7 and HepG2 and validated the results using the zebrafish in vivo model. PCSK9 deficiency led to strong inhibition of cell proliferation in all cell lines. At the lipid metabolic level, PCSK9 inhibition was translated by an increase in intracellular neutral lipids, phospholipids and polyunsaturated fatty acids as well as a higher accumulation of lipid hydroperoxide. Molecular signaling analysis involved the disruption of the sequestome 1/Kelch-like ECH-associated protein 1/nuclear factor erythroid 2-related factor 2 (p62/Keap1/Nrf2) antioxidative axis, leading to ferroptosis, for which morphological features were confirmed by electron and confocal microscopies. The anti-tumoral effects of PCSK9 deficiency were validated using xenograft experiments in zebrafish. The inhibition of PCSK9 was effective in disrupting the oncometabolic process, inducing metabolic exhaustion and enhancing the vulnerability of cancer cells to iron-triggered lipid peroxidation. We provide strong evidence supporting the drug repositioning of anti-PCSK9 approaches to treat liver cancers.

## 1. Introduction

Enhanced lipid metabolism in liver cancer is an important feature in the oncogenic process [1]. Interestingly, unlike most normal cells, even highly proliferative ones, their tumoral counterparts mostly display a preference to increased endogenous fatty acid (FA) biosynthesis in spite of dietary lipid abundancy [2]. This de novo pathway has a significant impact on the qualitative composition of the membranes via the enrichment of phospholipids with saturated and/or monounsaturated fatty acid chains since mammalian cells have a limited ability to synthesize polyunsaturated FAs (PUFAs) [3]. Because saturated FAs are less prone to lipid peroxidation than polyunsaturated ones, by shifting lipid acquisition from external sources toward de novo lipogenesis, cancer cells acquire specific protective features in their membranes to resist oxidative stress-induced cell insults. 

This context highlights the importance of the proprotein convertase subtilisin/kexin type 9, or PCSK9. Targeting PCSK9 to treat cancer becomes attractive not only because of its critical role in lipid metabolism but also because of its growing place in the recent literature, which has reported its tight association with the incidence and progression of several cancers [4]. Moreover, there is a diversity of therapeutic approaches targeting this enzyme that have been evaluated and, in some cases, approved for clinical use to lower cholesterol levels in patients suffering from hyperlipidemia.

Indeed, PCSK9 is a serine protease that belongs to the family of proprotein convertases, mainly involved in the degradation of the hepatic low-density lipoprotein receptor (LDLR) present on hepatocytes’ surface. This function interferes with the primary pathway of LDL-cholesterol (LDL-C) uptake from the circulation, thus leading to the increase in blood cholesterol levels. PCSK9 can target other members of LDLRs such as very low-density lipoprotein receptor (VLDLR), apolipoprotein E receptor 2 (ApoER2), cluster of differentiation 36 (CD36) and CD81 [5].

Because of its essential role in lipid metabolism, many therapeutic approaches targeting PCSK9 have been implemented in combination with other hypolipidemic drugs such as statins to treat patients with hyperlipidemia and cardiovascular diseases. 

Besides monoclonal antibodies (mAbs), one of the most recent approaches is the first-in-class chemically synthesized small interfering ribonucleic acid (siRNA) against PCSK9, inclisiran (Leqivo®; Novartis, Basel, Switzerland), which was approved for use in adults suffering from heterozygous familial hypercholesterolemia (FH) or non-familial and mixed dyslipidemia in Europe in late 2020 [6,7,8].

The expression of PCSK9 is deregulated in different types of cancers, including HCC neuroglioma, breast cancer, colorectal cancer and others [4,9], highlighting a major question about the therapeutic strategies that could be adopted to target PCSK9 in these cancers. More specifically, a very recent study reported the high expression of PCSK9 in tumor tissues in HCC patients. In this research, the expression of PCSK9 in tumors correlated with a poor prognosis after curative resection and was an independent risk factor for overall and disease-free survival. This illustrates the potential of PCSK9 as a prognostic marker for HCC [10].

The present study aims at assessing the interest of inhibiting PCSK9 by siRNA/shRNA (short hairpin RNA) in liver cancer using different cell lines and a tumor xenograft approach. We show the critical role of this enzyme in controlling the homeostasis of lipid biosynthesis and traffic but also and for the first time, its place in maintaining redox homeostasis. Interference with this protein leads to aberrant lipid metabolism, high lipid peroxidation and the death of liver cancer cells by ferroptosis.

## 2. Materials and Methods

### 2.1. Patient Samples

All patients were recruited in accordance with European and French laws and institutional ethical guidelines. Non-tumoral, tumoral and adjacent non-tumoral livers were collected from patients treated at French university hospitals and centralized by the French HEPATOBIO network.

### 2.2. Transcriptomic Data Acquisition

The R2: Genomics Analysis and Visualization Platform (http://r2.amc.nl, accessed on 23 September 2022 was used to generate the gene expression data from different available datasets. In this study, two different datasets were selected: Hepatoblastoma—López-Terrada—55- fRMA—u133p2 (GEO ID: gse75271) [11] and Tumor HCC—Wu—134—MAS50 (GEO ID: gse45436) [12]. Numeric data of gene expression were downloaded in Excel files and graphs were generated using GraphPad Prism 9 software (GraphPad Software, Inc., San Diego, CA, USA). In addition, the level of expression of lipid-related genes in the hepatic cell lines was generated by referring to the transcriptomic data obtained on these cell lines by Hooks et al. [13]. 

### 2.3. RNA Sequencing Analysis

Total RNA from Huh7 and THLE-2 cell lines was extracted using the *mir*Vana kit (Thermo Fisher Scientific, Waltham, MA, USA) according to the supplier’s protocol and the analysis was conducted by Hooks et al. [13] in a manner similar to what they performed for the other cell lines and patient tissues.

### 2.4. Cell Culture 

Human HCC (Huh7) and hepatoblastoma (HB) (HepG2, Huh6) derived cell lines were cultured in Dulbecco’s Modified Eagle Medium (DMEM GlutaMAX™, Gibco, Thermo Fisher Scientific, Waltham, MA, USA, supplemented with high (4.5 g/L) for Huh7 and HepG2 or low (1 g/L) D-glucose for Huh6) supplemented with 10% fetal bovine serum (FBS), 100 µg/mL streptomycin and 100 U/mL penicillin. The cells were maintained at 37 °C in a humidified atmosphere of 5% CO_2_. Cell line authentication was performed in April 2021 using short tandem repeats (LGC, Molsheim, France) and the absence of mycoplasma contamination was tested on a monthly basis.

### 2.5. Lentivirus Production and Transduction

Lentivirus vector production was performed using the Vect’UB service platform (INSERM US 005-CNRS UMS 3427-TBM-Core, Université de Bordeaux, Bordeaux, France). Lentiviral particles were produced by transient transfection of human embryonic kidney cells (HEK293T) according to standard protocols. In brief, subconfluent HEK293T cells were co-transfected with lentiviral genome (psPAX2) (a gift from Didier Trono (Addgene plasmid # 12260)), with an envelope-coding plasmid (pMD2G-VSVG) and with vector constructs (305 pLKO-sh886 or 306 pLKO-shCTR) by calcium phosphate precipitation. LVs were harvested 48 h post-transfection and concentrated by ultrafiltration. Viral titers of VSV-g pseudotype pLV lentivectors were determined by transducing the HEK293T cells with serial dilutions of viral supernatant and lentiviral integration was evaluated by quantitative PCR using RRE primers. The following forward (F) and reverse (R) sequences of shPCSK9-886 were used:

F-5′ CCGGGGGTCATGGTCACCGACTTCG*CTCGAG*CGAAGTCGGTGACCATGACCCTTTTT-3′ and R-5′ AATTCAAAAAGGGTCATGGTCACCGACTTCG*CTCGAG*CGAAGTCGGTGACCATGACCC 3′. The hairpin sequence of negative control shRNA is:

CCTAAGGTTAAGTCGCCCTCGCTCGAGCGAGGGCGACTTAACCTTAGG (http://www.addgene.org/pgvec1?f=c&identifier=1864&atqx=plko&cmd=findpl, accessed on 15 January 2021).

HepG2 cells stably expressing a tomato transgene were generated by lentivirus transduction at a multiplicity of infection (MOI) of 10 with an MND-Tomato-265 virus (donated by the Vect’UB platform). Red fluorescent cells were sorted by Fluorescence-Activated Cell Sorting (FACS). Stable inhibition of PCSK9 expression was induced by cell transduction with the lentivirus 305 pLKO-sh886 (shPCSK9) or the control (306 pLKO-shCTR) at an MOI of 10. Transduced cells were selected using puromycin (P8833, Sigma-Aldrich, St. Louis, MO, USA) at 3 μg/mL.

### 2.6. siRNA Transfection

Small interfering siRNAs (si1 {sense: 5′ GUGCUCAACUGCCAAGGGA[dT][dT] 3′; anti-sense: 5′ UCCCUUGGCAGUUGAGCAC[dT][dT] 3′} and si2 {sense: 5′ GGGUCAUGGUCACCGACUU[dT][dT] 3′; anti-sense: 5′ AAGUCGGUGACCAUGACCC[dT][dT] 3′}) against PCSK9 (Sigma-Aldrich, St. Louis, MO, USA) were diluted in 1X siMAX dilution buffer (30 mM HEPES, 100 mM KCl, 1 mM MgCl_2_, pH 7.3, Eurofins, Luxembourg). Hepatic cancer cells were transfected independently with 20 nM si1 or 2 or control siCTR (AllStars Negative Control siRNA, Qiagen, Hilden, Germany) using a lipofectamine RNAi MAX transfection reagent (Invitrogen, Waltham, MA, USA) according to the manufacturer’s instructions for reverse transfection. For transfection, lipofectamine RNAi MAX was diluted by 1/100th in a transfection medium (OptiMEM, Gibco^TM^, Thermo Fisher Scientific, Waltham, MA, USA). 

### 2.7. Proliferation Assay

Cells were transfected with siRNAs for 24 h before seeding them into 96-well plates in triplicates at various densities (3000 cells/well for Huh7, 2000–2500 cells/well for HepG2, 700–2000 cells/well for Huh6) and then treated with various concentrations of ferrostatin-1 (0–20 µM) or α-tocopherol (0–200 µM). The experiment was followed for different time intervals, during which a fresh medium with the drug was changed every 2 days. The proliferation of cells was assessed using CellTiter 96® AQ_ueous_ One Solution Reagent (Promega, Madison, WI, USA) and the absorbance was recorded at 490 nm using ClarioStar (BMG Labtech, Champigny-sur-Marne, France).

### 2.8. Western Blot

Cells were lysed in RIPA buffer (Sigma-Aldrich, St. Louis, MO, USA) supplemented with protease and phosphatase inhibitor cocktails (Roche, Basel, Switzerland) and centrifuged at 13,000 rpm for 15 min at 4 °C. Protein concentration was determined using the Pierce™ BCA protein assay kit (Thermo Fisher Scientific, Waltham, MA, USA). Approximately 40 μg of proteins were loaded per lane for Western blot analyses in 4–15% precast polyacrylamide gel (Bio-Rad, Hercules, CA, USA) and blotted onto 0.2 μm nitrocellulose membrane (Bio-Rad, Hercules, CA, USA). The membranes were blocked in 5% BSA in TBST (20 mM Tris, 150 mM NaCl, 0.1% Tween 20), then incubated with each of the following specific primary antibodies: a sheep anti-PCSK9 (1 μg/mL, AF3888, R&D Systems, Minneapolis, MN, USA), ferroptosis antibody sampler kit (Cell signaling, 29650), rabbit anti-HO-1/HMOX1 (1:3000, 10701-1-AP, ProteinTech Group, Inc., Rosemont, IL, USA), mouse anti-NQO1 (1:7000, 67240-1-Ig, ProteinTech Group, Inc., Rosemont, IL, USA), mouse anti-GAPDH HRP conjugated (1:10,000, BLE649203, BioLegend, San Diego, CA, USA) and goat anti-vinculin (1:1000, sc-7649, Santa Cruz Biotechnology, Dallas, TX, USA) overnight at 4 °C. After incubation with the appropriate secondary antibody coupled with horseradish peroxidase (goat anti-mouse IgG (H+L) HRP, 1:3000, 170-6516, Bio-Rad; rabbit anti-goat IgG HRP, 1:1000, HAF017, R&D Systems; goat anti-rabbit IgG HRP, 1:5000, A0545, Sigma-Aldrich; rabbit anti-sheep IgG HRP, 1:3000, 402100, Calbiochem, San Diego, CA, USA), all blots were revealed with Fusion FX (Vilber Lourmat, Marne-la-Vallée, France) following incubation with the ECL reagents from Bio-Rad. Quantification was performed using ImageJ software (National Institutes of Health, Bethesda, MD, USA).

### 2.9. Lipidomics 

The extraction of lipids from cell pellets was performed using chloroform:methanol (2:1, *v*/*v*), following the original Folch method, with a CHCl_3_:MeOH:H_2_O ratio of 8:4:3 (*v*/*v*). For this purpose, 0.5 mL methanol and 1 mL chloroform were added directly to the cell pellet. The suspension was incubated for 30 min on ice with repeated vortexing. After the addition of water to separate the aqueous and organic phases, the mixture was incubated on ice for an additional 10 min. The samples were then centrifuged at 1000× *g* for 5 min. The organic phase was transferred to a new tube. The aqueous layer was re-extracted with 2 mL chloroform:methanol (2:1, *v*/*v*). The chloroform layers were combined, evaporated to dryness and resuspended in 100 µL chloroform:methanol (1:1, *v*/*v*).

Phospholipids were analyzed by loading 25 µL of total lipids onto HPTLC plates (60F254, Merck, Darmstadt, Germany) and developed with methyl acetate/n-propanol/chloroform/methanol/0.25% aqueous KCl (5:5:5:2:1.8, *v*/*v*) as solvent. Neutral lipids were analyzed by loading 25 µL of total lipids onto HPTLC plates and developed with hexane/ethyl ether/formic acid (10:5:0.5, *v*/*v*).

For lipid quantification, the plates were then immersed in a copper acetate solution (3% copper acid + 8% phosphoric acid in distilled water) and heated at 115 °C for 30 min. Lipids were identified by co-migration with known standards and quantified by densitometric analysis using a TLC scanner (CAMAG, Muttenz, Switzerland).

### 2.10. Radiolabeling Experiment

For radiolabeling experiments, the counted cells of each sample were transferred to a glass tube in 6 mL of DMEM medium. To start the reaction, 200 nmol (10 µCi) of [1-^14^C] acetate (PerkinElmer Life Sciences, Waltham, MA, USA) were added to each tube and the tubes were incubated at 37 °C in 5% CO_2_. The uptake of acetate was studied for each sample at 3 different time points (1 h, 2 h and 4 h). To stop the reaction, the samples were centrifuged at 1000× *g* for 5 min and the supernatants were removed. After the addition of 2 mL chloroform/methanol (2:1, *v*/*v*), the cells were incubated overnight at −20 °C. To separate the aqueous and organic phases, 1 mL of 0.9% NaCl was added and the mixtures were centrifuged at 1000× *g* for 5 min. The organic phases were transferred to a new tube. The aqueous layer was re-extracted with 2 mL chloroform/methanol (2:1, *v*/*v*). The chloroform layers were combined and washed one time with 1 mL 0.9% NaCl. The organic phases were evaporated to dryness, resuspended in 100 µL chloroform/methanol (2:1, *v*/*v*) and stored at −20 °C. Radiolabeled products were analyzed by thin-layer chromatography using HPTLC Silica Gel 60 plates (Merck, Darmstadt, Germany). 

### 2.11. FA Saturation and Lipid Peroxide Analysis 

Cell pellets of counted cells were directly used for fatty acid analysis. Fatty acid methyl esters were obtained by transmethylation at 90 °C for 1 h with 0.5 M sulfuric acid in methanol containing 2% (*v*/*v*) dimethoxypropane and 50 μg of heptadecanoic acid (C17:0) as internal standards. After cooling, 1 mL of NaCl (2.5%, *w/v*) was added and fatty acyl chains were extracted with 1 mL hexane. Samples were subsequently analyzed by GC-MS as described by Domergue et al. [14]. The measurement of lipid hydroperoxide was performed using the Lipid Hydroperoxide (LPO) Assay Kit (Cayman Chemical, Ann Arbor, MI, USA) as instructed by the manufacturer. 

### 2.12. Immunohistochemistry (IHC)

The 3.5-µm thick sections of hepatoblastoma tumors were deparaffinized, rehydrated and antigen retrieval was performed in 0.01 M citrate buffer pH 6 solution. All staining procedures were performed by an autostainer (Dako-Agilent, Santa Clara, CA, USA) using standard reagents provided by the manufacturer. The sections were blocked using EnVision™ Flex peroxidase-blocking reagent (SM801, Dako-Agilent) to block endogenous peroxidase, then washed and incubated with rabbit anti-PCSK9 (1:100, 55206-1-AP, ProteinTech Group, Inc., Rosemont, IL, USA). Incubation in horseradish peroxidase (EnVision Flex/HRP, SM802, Dako-Agilent, Santa Clara, CA, USA) was used for signal amplification. A 3,3′-diaminobenzidine (DAB, Dako-Agilent, Santa Clara, CA, USA) development was used for detecting primary antibodies by producing a crisp brown end product at the site of the target antigen. The slides were counterstained with hematoxylin, dehydrated and mounted. Each immunohistochemical run contained a negative control (buffer, no primary antibody). Sections were visualized with a Hamamatsu NANOZOOMER 2.0 HT at 20× magnification in the Photonic Unit of the Bordeaux Imaging Center (BIC). 

### 2.13. Transmission Electron Microscopy (TEM)

HepG2 cells transduced with shCTR and shPCSK9 were seeded in a Nunc™ Lab-Tek™ 8-chamber slide system (Thermo Fisher Scientific, Waltham, MA, USA) to a confluence of 80%. The cells were fixed with 2.5% (*v*/*v*) glutaraldehyde and 4% (*v*/*v*) paraformaldehyde in 0.1 M phosphate buffer (pH 7.4) during 2 h at room temperature (RT), washed in 0.1 M phosphate buffer (pH 7.4) and then postfixed in 1% osmium tetroxide in water during 1 h. Then, samples were washed in water, dehydrated through a series of graded ethanol and embedded in a mixture of pure ethanol and epoxy resin (Epon 812; Delta Microscopy, Toulouse, France) 50/50 (*v*/*v*) during 2 h and then in 100% resin overnight at RT. The polymerization of the resin was carried out over a period of 48 h at 60 °C. Samples were then sectioned using a diamond knife (Diatome, Biel-Bienne, Switzerland) on an ultramicrotome (EM UC7, Leica Microsystems, Vienna, Austria). Ultrathin sections (70 nm) were picked up on copper grids. Grids were examined with a transmission electron microscope (H7650, Hitachi, Tokyo, Japan) at 80 kV.

### 2.14. In Vivo Zebrafish Model

Zebrafish were maintained at 28 °C and in light cycle conditions (12 h). The *Casper* mutant fish line was purchased from the Zebrafish International Resource Center (ZIRC). For zebrafish xenotransplantation, 48 hours post-fertilization (hpf) zebrafish embryos were dechorionated and anesthetized in egg water solution containing 0.04 mg/mL tricaine (Sigma-Aldrich, St. Louis, MO, USA) before human cell injection. Approximately 200 to 500 fluorescent cells were injected (Eppendorf® Femtojet® microinjector) into the ducts of Cuvier of each embryo, and the zebrafish were maintained in 0.3X Danieau’s solution for 1 h at 28 °C. After confirmation of a visible cell mass at the injection site, the zebrafish were transferred to a 24-well plate in 500 μL of a 0.3X Danieau’s solution incubator and maintained at 34 °C. The zebrafish with already formed metastasis at 1 hour post-injection (hpi) were discarded. 

After 24 hpi and 48 hpi, living zebrafish embryos were anesthetized using 0.04 mg/mL tricaine and observed under an inverted fluorescence microscope (Nikon Eclipse TS100). Low magnification (X4 objective) was used to provide an overview of the tumor cell metastasis pattern throughout the fish. Pictures were taken by using Archimed (Microvision Instruments) software. Fiji software was used for automated tumor area evaluation. Briefly, a 30–225-intensity threshold was set to select cells and the ‘Analyze’ particle tool was used with default selection of the cell size and cell shape during counting. A Fiji macro was generated using the ‘Record’ function to streamline the analyses. 

### 2.15. Statistical Analysis

Statistical analyses were performed using GraphPad Prism 9 software (GraphPad Software, Inc., San Diego, CA, USA). For two-group comparison, we used the *t-*test when values were ≥15; otherwise, the Mann–Whitney rank-sum test was used. For quantitative comparisons of more than two samples, the one-way ANOVA test was used followed by the Bonferroni post hoc test. Two-way ANOVA followed by the Bonferroni post hoc test was used for experiments containing three or more groups at different time points. For correlation graphs, a two-tailed Pearson correlation test was used. The experiments were carried out independently at least 3 times unless otherwise stated and each time, we included 3 technical replicates. In this case, n = number of independent experiments. A *p*-value of < 0.05 was considered to be statistically significant. For all data in figures, *: *p* < 0.05, **: *p* < 0.01, ***: *p* < 0.001, ****: *p* < 0.0001 or exact *p*-values were indicated. All tests were two-sided.

## 3. Results

### 3.1. PCSK9 Is Overexpressed in Liver Cancers

**Figure 1:**
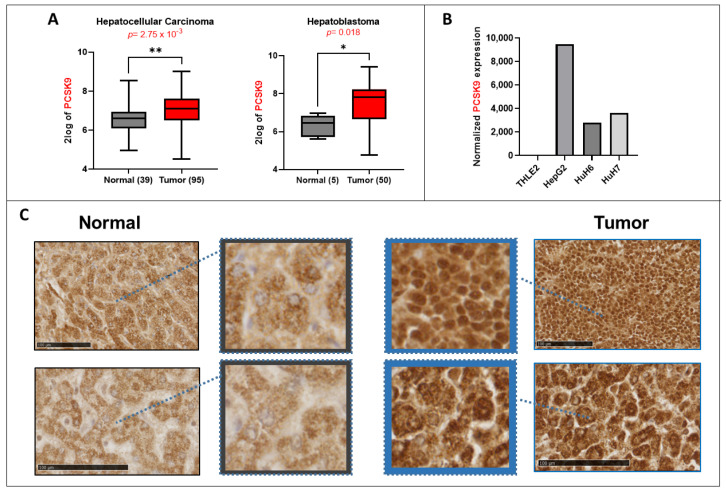
**Lipid metabolism in liver cancer.** (**A**) *PCSK9* gene expression upregulation in 2 transcriptomic datasets generated from the R2: Genomics Analysis and Visualization Platform (http://r2.amc.nl, accessed on 23 September 2022). Left histogram, hepatocellular carcinoma (HCC): Wu—134—MAS 5.0—u133p2. Right histogram, hepatoblastoma (HB): López-Terrada—55-fRMA—u133p2. Unpaired *t*-test, * *p* < 0.05; ** *p* < 0.01. (**B**) Normalized gene expression of *PCSK9* in three liver cancer cell lines (HepG2, Huh6 and Huh7) and one normal cell line (TLHE2). (**C**) PCSK9 staining of HB tumoral tissues was compared to normal liver tissues from the same patients. PCSK9 immunostaining of the whole tissue sections is shown in included subsets in which blue squares and arrows indicate the depicted enlarged area of staining. Samples were collected as described in the Materials and Methods section. The black bars represent 100 µm.

Using available transcriptomic databases, we showed elevated gene expression of PCSK9. It is significantly upregulated in both adult (HCC) and pediatric (HB) liver cancers according to the data from Wu et al. and Lopez-Terrada et al., posted in the R2: Genomics Analysis and Visualization Platform (Figure 1A). We confirmed the same tendencies in three liver cancer cell lines (e.g., HepG2, Huh6 and Huh7) in comparison with the normal liver cell line THLE2 (Figure 1B). IHC analyses of PCSK9 in tumoral tissues showed mostly high expression levels (Figure 1C). However, we noticed more heterogeneous expression patterns and unlike the rather diffuse cytoplasmic expression of PCSK9 observed in the normal liver, the expression in tumoral tissues appeared to be more perinuclear and nuclear (Figure 1C). 

Hence, according to the analysis of public databases and our own assessment, PCSK9 expression is higher in liver cancer tissues and cells. Intriguingly, PCSK9 seems to localize into the nucleus of liver cancer cells, which has never been reported before, underlining some differential and a new type of regulation and impact of this enzyme in the context of liver cancer. We next investigated how the inhibition of PCSK9 would affect the whole oncogenic process.

### 3.2. Targeting of PCSK9 Using siRNA Inhibits Cell Growth in Cancer Cells

**Figure 2:**
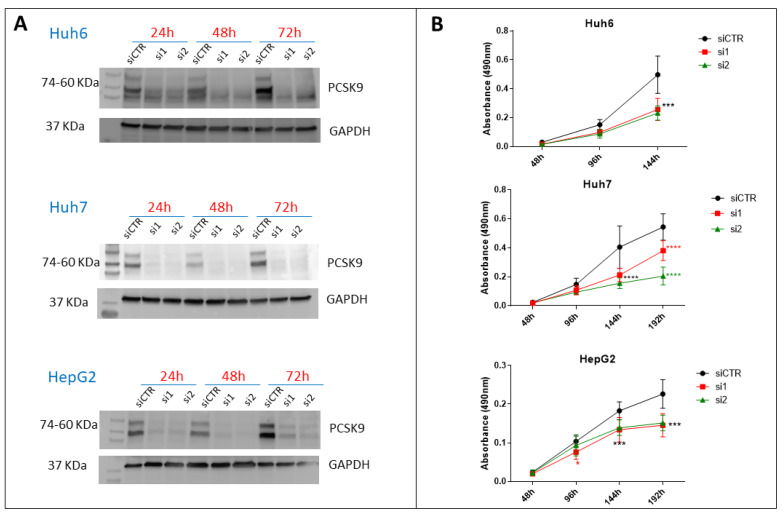
**Effect of PCSK9 silencing by siRNA on liver cancer cell proliferation.** (**A**) Validation of the depletion of PCSK9 by siRNA silencing. Cell extracts were prepared 24, 48 and 72 h after transfection with a control siRNA (siCTR) or targeting PCSK9 (si1 and si2) of Huh6, Huh7 or HepG2 cells. A total of 40 µg of cell proteins were loaded per lane on an SDS-PAGE. After electrophoresis and transfer, the membrane was analyzed by Western blot using anti-PCSK9 and GAPDH antibodies for comparison of loading. The two bands observed for PCSK9 correspond to the pro-PCSK9 (74 kDa) and cleaved PCSK9 (60 kDa). (**B**) Evaluation of liver cancer cell proliferation by MTS after transfection with si1 and si2 in comparison with siCTR. Two-way ANOVA test, *** *p* < 0.001; **** *p* < 0.0001 (n = 3, with 3 technical replicates within each biological replicate).

For the specific targeting of PCSK9, we designed two different specific sequences of siRNA (si1 and si2), which were validated for PCSK9 inhibition by Western blot (Figure 2A). Three days after transfection, 96% and 99% of PCSK9 were lost in Huh7 and Huh6, respectively, with the two tested siRNAs, while in HepG2, 87% and 95% of PCSK9 were lost with si1 and si2, respectively. Next, we used this approach to test the effect of PCSK9 inhibition on cell proliferation (Figure 2B). Transfection with both siRNAs was comparatively effective in slowing down the proliferation of all cell lines tested, with some fluctuations in Huh7, where si2 exhibited a stronger inhibition effect than si1 (Figure 2B).

### 3.3. PCSK9 Silencing Disrupts Lipid Metabolism in Cancer Cells

**Figure 3:**
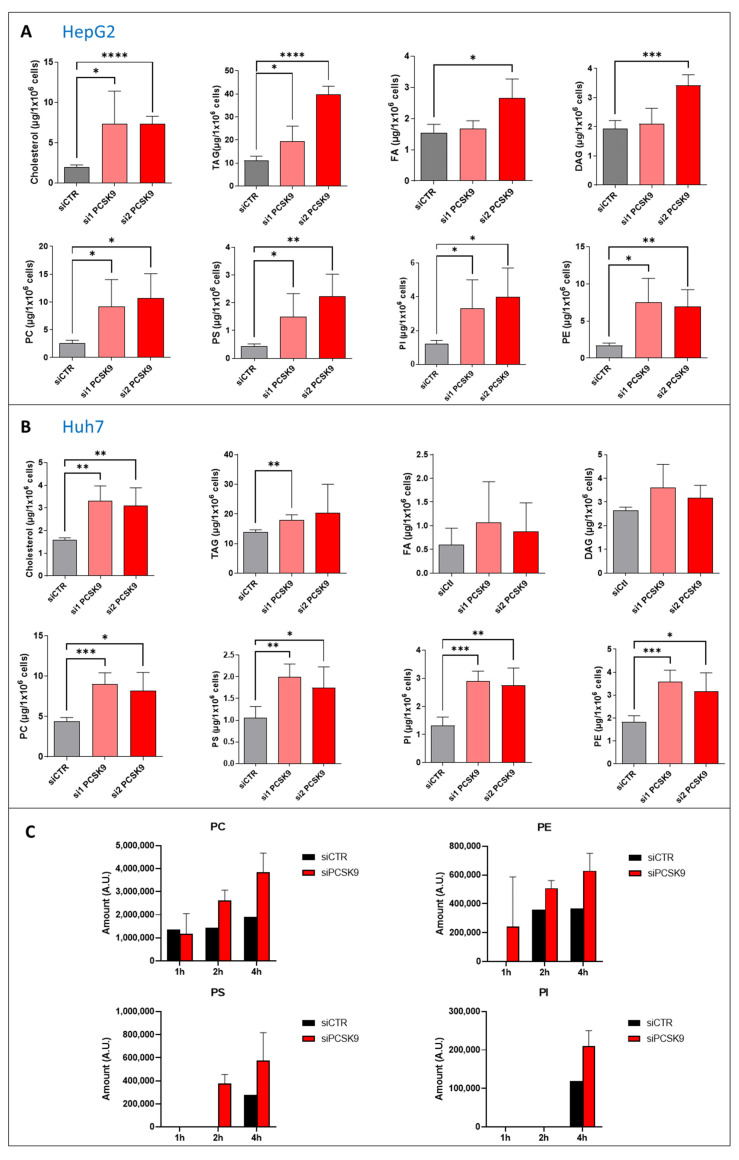
**Quantification of neutral lipids and phospholipids after PCSK9 silencing.** (**A**,**B**) Cholesterol, triacyl glycerol (TAG), fatty acid (FA) diacyl glycerol (DAG), phosphatidylcholine (PC), -serine (PS), -inositol (PI) and -ethanolamine (PE) were measured in (**A**) HepG2 and (**B**) Huh7 after transfection with PCSK9 siRNA or the control for 144 h. Unpaired *t*-test where each siRNA PCSK9 group was compared to the CTR group alone, * *p* < 0.05; ** *p* < 0.01; *** *p* < 0.001; **** *p* < 0.0001 (n = 3, with 3 technical replicates within each biological replicate). (**C**) Cells were transfected with siRNA (si1 and si2) for 72 h then fed with [1-^14^C] acetate. Lipid extraction was performed 1, 2 and 4 h after [1-^14^C] acetate feeding and radiolabeled PC, PE, PS and PI amounts were measured at each time point. Average values were obtained by combining values from both PCSK9 siRNAs (n = 1, with 3 technical replicates within each condition).

PCSK9 is essentially involved in maintaining adequate cellular lipid fluxes by modulating several lipid receptors at the cell surface. Because of this important role in lipid metabolism, we quantified major neutral lipid species and phospholipids in Huh7 and HepG2 cells in the presence or absence of PCSK9 expression. The extent of changes in lipid amounts among both types of cells was very important (Figure 3A,B). We observed some variations between both the siRNA and cell types. Nonetheless, most of the lipid entities were significantly higher in PCSK9-deficient cells, with a 3- to 5-fold increase for some lipids such as the cholesterol and phospholipids in the HepG2 cells (Figure 3A). These data highlight a major disruption of lipid homeostasis with increased intracellular lipids which may be the consequences of sparing most lipoprotein receptors normally degraded by PCSK9. To find out whether the inhibition of PCSK9 was affecting lipogenesis as well, we conducted a radioactive ^14^C tracing experiment of the metabolites by feeding HepG2 cells with ^14^C-labeled acetate for 1, 2 and 4 h. Lipidomic analyses was then performed to measure the amount of phospholipids, including phosphatidylserine (PS), phosphatidylinositol (PI), phosphatidylethanolamine (PE) and phosphatidylcholine (PC) (Figure 3C). Phospholipid analysis showed very quick enrichment of PC under both conditions (e.g., 1 h after treatment). However, cells lacking PCSK9 accumulated more PC starting 2 h after acetate feeding. PE enrichment started one hour after acetate feeding in cells treated with siPCSK9. No detectable PE was seen at this time point in siCTR-transfected cells. The enrichment of PE was seen in the control cells only after 2 h of acetate feeding and stayed constant over time but below its levels in cells lacking PCSK9. As for PS, the enrichment was slower than PE but again faster in the absence of PCSK9, appearing first in these cells after 2 h of feeding with acetate. Finally, PI enrichment appeared later (e.g., at 4 h) but again in lower amounts in the control cells. Globally, the acetate tracing experiment showed more active synthesis of phospholipids reflecting enhanced lipogenesis in cells silenced for PCSK9, suggesting that intracellular lipid accumulation in these cells is originated not only from an increased uptake but also from endogenous lipid synthesis. After these quantitative changes in cellular lipids, we wondered whether the saturation level of fatty acids was affected by PCSK9 status.

### 3.4. PCSK9 Silencing Leads to Excessive Accumulation of Lipids in Cancer Cells

**Figure 4:**
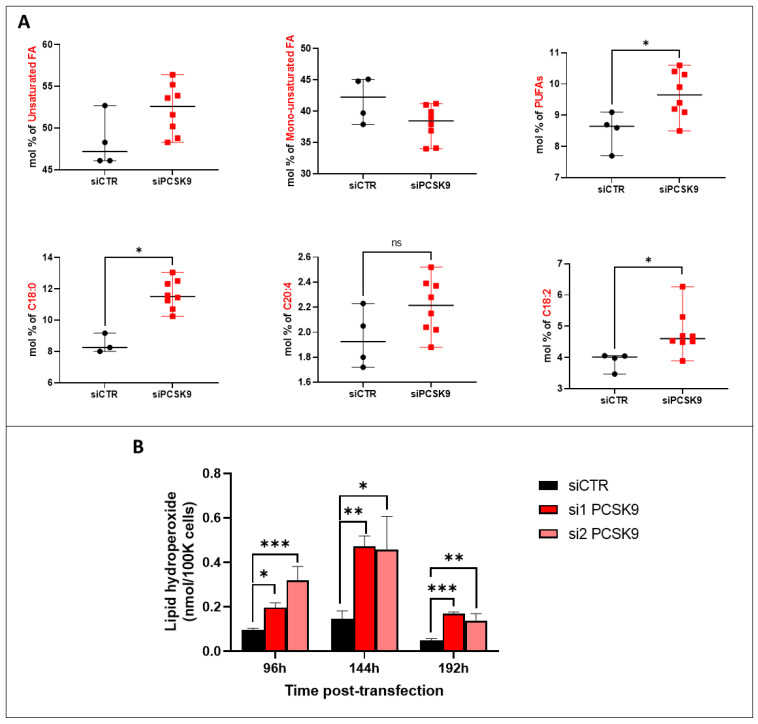
**Fatty acid unsaturation and lipid peroxidation after PCSK9 silencing.** (**A**) FA composition and saturation in HepG2 cells silenced or not for PCSK9. Mann–Whitney *t*-test (n = 3). (**B**) Lipid hydroperoxide quantification in HepG2 cells after transfection with PCSK9 or control siRNA (si1 and si2 or siCTR) for 96 h, 144 h and 192 h. Quantities are represented in nmol per 100,000 cells. Ordinary one-way ANOVA test. * *p* < 0.05; ** *p* < 0.01; *** *p* < 0.001 (n = 3, with 3 technical replicates within each biological replicate).

By examining the saturation levels of the FAs (Figure 4A), we observed a borderline increase in total unsaturated FA (*p* = 0.06) after siRNA transfection, although the increase was significant for PUFA and C18:2 FA as an example. The increase in PUFA amounts in the absence of PCSK9 probably resulted from an enhanced uptake of external sources of lipids, thereby leading to the enrichment in peroxidation-prone intracellular FAs [3]. In order to verify whether lipid peroxidation was enhanced in this case, we measured the levels of lipid hydroperoxide in HepG2 cells 4, 6 and 8 days after transfection with PCSK9 siRNA or control siRNA (Figure 4B). Indeed, silencing PCSK9 led to a significant increase in the amount of lipid hydroperoxide at all time points which is probably a consequence of the presence of higher oxidation-sensitive PUFAs in the absence of PCSK9. Globally, when PCSK9 is silenced, cells seem to be gearing up all of their machinery to take up more lipids from the environment, generating quantitative and qualitative imbalances in intracellular lipids. 

### 3.5. PCSK9 Targeting Inhibits the Anti-Oxidative p62/Keap1/Nrf2 Pathway Triggering Ferroptosis

**Figure 5:**
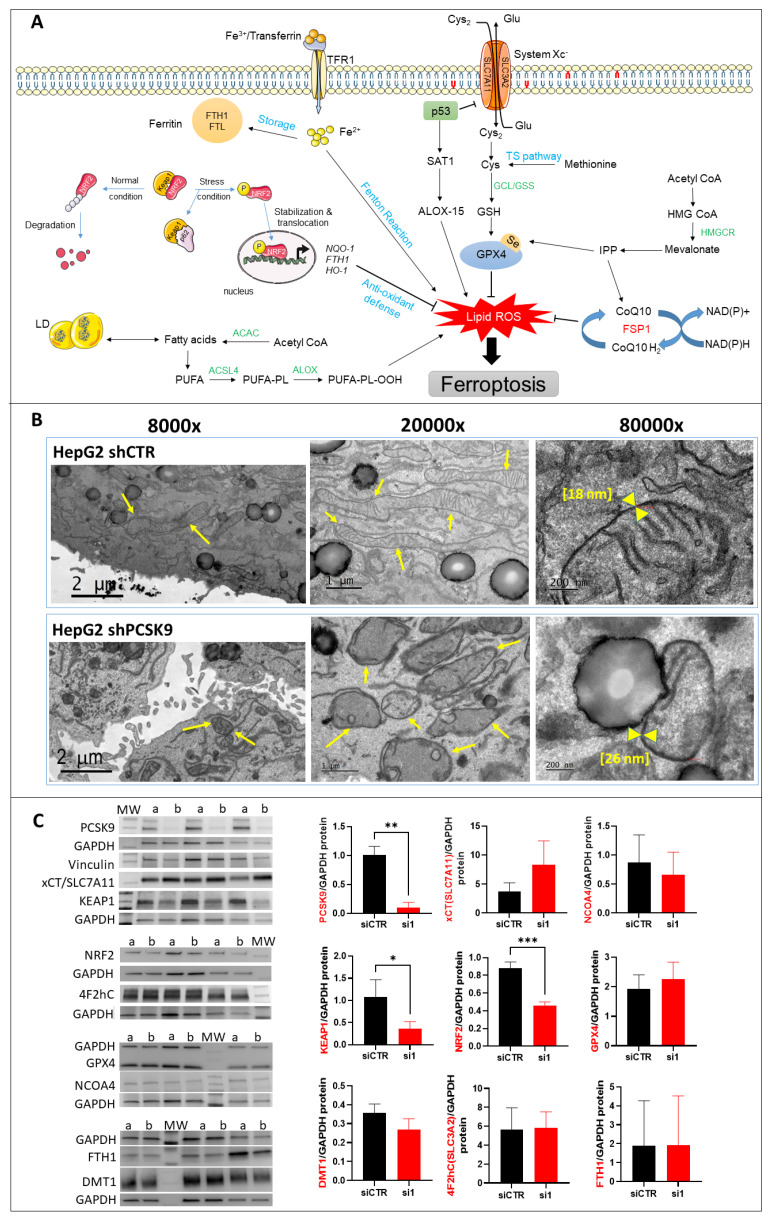

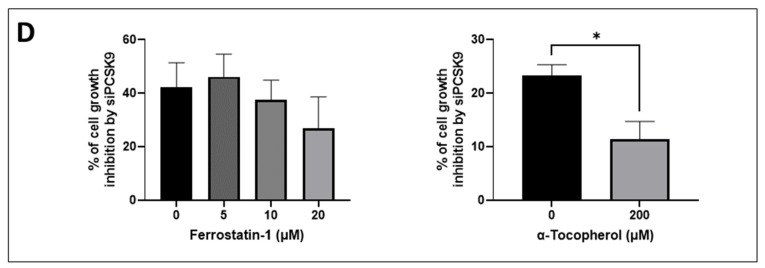
**Morphological and molecular features of ferroptosis with PCSK9 deficiency.** (**A**) The central regulator that inhibits ferroptosis is the selenoprotein GPX4, which together with reduced glutathione (GSH), has an antioxidant capacity against lipid reactive oxygen species (ROS), thus blocking ferroptosis (right side). The pathways that lead to the activation and synthesis of GPX4/GSH and hence ferroptosis inhibition include the System Xc^-^-mediated import of cysteine (Cys), production of cysteine by the transsulfuration (TS) pathway and finally, the production of selenocysteine (Se) by the mevalonate pathway. Another important product of the mevalonate pathway is the CoQ10, which can inhibit ferroptosis independently of GPX4. The oxidoreductase FSP1 reduces CoQ10 to ubiquinol (CoQ10 H_2_) which can trap lipid ROS and then regenerates CoQ10 using NAD(P)H. (left side) The p62/Keap1/Nrf2 pathway plays an important role in inhibiting ferroptosis by activating the expression of antioxidant defense genes NAD(P)H quinone dehydrogenase 1 (NQO-1), FTH-1 and heme oxygenase 1 (HO-1). Under normal conditions, Keap1 binding to Nrf2 induces its ubiquitination and hence proteasome degradation. Under stress conditions, p62 binds to Keap1, the inhibitor of Nrf2, thus stabilizing Nrf2 which can then translocate to the nucleus and induce the expression of antioxidant defense genes. On the other hand, the pathways that lead to lipid ROS accumulation and induction of ferroptosis include: the accumulation of free intracellular ferrous (Fe2^+^) that can produce hydroxyl and peroxide radicals by the Fenton reaction, hence oxidizing lipids, and tumor protein p53 (p53) activation that inhibits system Xc^−^ uptake of cystine by decreasing the expression of solute carrier family 7 member 11 (SLC7A11), hence affecting GSH/GPX4 antioxidant capacity. It can also activate SAT1 transcription factor to induce lipid peroxidation by increasing ALOX-15 levels; this leads to the production of PUFAs, which are sensitive to lipid peroxidation, resulting in ferroptosis. *Abbreviations: ACAC: Acetyl-CoA carboxylase; ACSL4: long-chain fatty Acyl-CoA synthetase 4; Fe3^+^: ferric cation; FTL: ferritin light chain; GCL: glutamate-cysteine ligase; GSS: glutathione synthetase; HMGCR: 3-hydroxy-3-methylglutaryl-CoA reductase; IPP: isopentenyl pyrophosphate; LD: lipid droplets, PL: phospholipid.* (**B**) TEM photomicrographs of HepG2 cells transduced with PCSK9 shRNA (top panels) and control shRNA (bottom panels) at different magnifications. The arrows point to the mitochondria. The mitochondrial membranes were measured at the higher magnification of 80,000 (between the yellow head arrows) and the results are indicated within brackets. (**C**) Western blot analyses of proteins involved in different signaling pathways of ferroptosis. Membrane images of each protein and their relative housekeeping genes were combined/fused for comparison purposes. Wells were loaded alternatively with samples from (a) siCTR- and (b) siPCSK9-transfected cells (from left to right). MW stands for molecular weight markers. GAPDH or vinculin were used for protein normalization. Unpaired *t*-test, * *p* < 0,05; ** *p* < 0,01, *** *p* < 0.001 (n = 3). (**D**) Histograms showing the percentage of cell growth inhibition induced by siPCSK9 in HepG2 cells normalized to siCTR, which was followed 6 days after treatment with ferrostatin-1 (left panel) and 8 days after treatment with α-tocopherol (right panel). Average values were obtained by combining values from both PCSK9 siRNAs. Unpaired *t*-test, * *p* < 0,05 (n = 1, with 3 technical replicates.).

In the absence of any confirmed sign of cell death by apoptosis/necrosis and senescence (data not shown), we investigated the features of other mechanisms. Taking into account the lipid phenotype of PCSK9 inhibition, the high level of lipid hydroperoxide and the possible induction of cell toxicity, we turned our attention to the potential occurrence of lipid peroxide-triggered cell death by ferroptosis (Figure 5A). Electron microscopy observations revealed obvious changes in the mitochondrial morphology consisting of size shrinkage, thickening of the double bilayer membranes and disappearance of mitochondrial cristae (Figure 5B). This last observation was confirmed by measuring the thickness of the mitochondrial double membrane, which was larger in some liver cells lacking PCSK9 (Figure 5B, right panels). 

To investigate the potential occurrence of ferroptosis at the molecular level, we assessed the changes in some proteins of the main cell signaling pathways described for this process (e.g., the System Xc^−^/glutathione peroxidase 4 (*Xc-/GPX4*), mevalonate (*MVA*), the sulfur transfer pathway, the p62-Keap1-Nrf2 pathway, the p53/solute carrier family 7 member 11 (*p53/SLC7A11*), the autophagy related 5-autophagy related 7-nuclear receptor coactivator 4 (*ATG5-ATG7-NCOA4*) pathway, the p53-spermidine/spermine N1-acetyltransferase 1-arachidonate 15-lipoxygenase (*p53-SAT1-ALOX15*) pathway and the heat shock protein beta 1-telomeric repeat factor 1-ferroptosis suppressor protein 1-ubiquinone/coenzyme Q10-nicotinamide adenine dinucleotide (phosphate) hydrogen (*HSPB1-TRF1, FSP1-COQ10-NAD(P)H*) pathway) (Figure 5A). Western blot analyses of protein extracts from cells transfected by one of the PCSK9 specific siRNAs, si1 and the control siRNA were performed on the following proteins: xCT/SLC7A11, GPX4, Keap1, Nrf2, ferritin heavy chain 1 (FTH1), NCOA4, solute carrier family 3 member 2 (4F2hC/CD98/SLC3A2) and divalent metal transporter (DMT1) (Figure 5C). The only significant changes were observed in the Keap1 and Nrf2 of the p62/Keap1/Nrf2 pathway with a 2.75-fold decrease for Keap1 (*p* = 0.04) and a 2-fold decrease for Nrf2 (*p* = 0.0008) in cells transfected with PCSK9 siRNA as compared to the controls. 

To further strengthen the link to ferroptosis, we used two inhibitors (antioxidants) of ferroptosis (e.g., ferrostatin-1 and alpha-tocopherol) (Figure 5D). As expected, cotreatment with α-tocopherol resulted in a strong reduction of the inhibitory effect of siPCSK9 on cell growth by 40%. As for ferrostatin-1, we noticed non-significant tendencies of dose-dependent decreases. It is important to point out that we did not expect a full recovery of cell proliferation after either of these treatments. Indeed, the anti-tumoral effect of anti-PCSK9 was mediated not only via triggering ferroptosis but also by its regulation of lipid fluxes and metabolism.

### 3.6. Blocking PCSK9 Has Anti-Tumoral Effects in Zebrafish In Vivo Model

**Figure 6:**
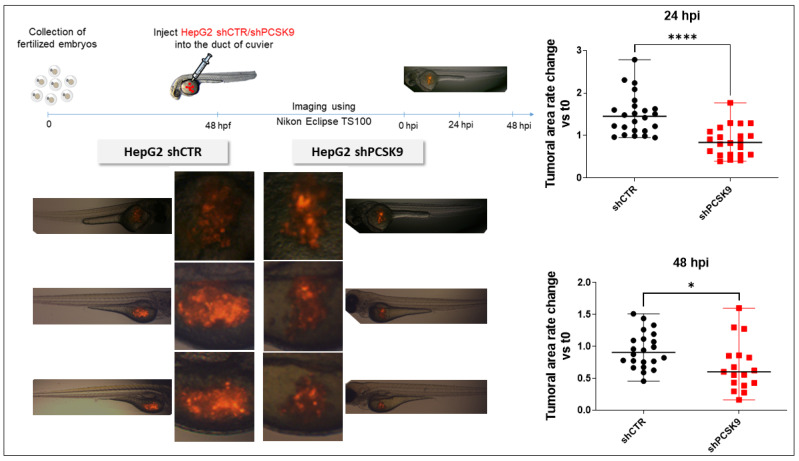
**Depleting PCSK9 impairs liver tumor development in vivo.** In zebrafish experiments: tomato red fluorescent labeled HepG2 with shPCSK9 or the control were xenografted in zebrafish embryos. Tumoral cell growth was evaluated by the quantification of the fluorescence 24 and 48 h post-injection (hpi). Unpaired *t*-test, *: *p* < 0.05, ****: *p* < 0.0001.

To validate the effect of blocking PCSK9 in vivo, we used an established model, that is, the zebrafish model xenografted with HepG2 labeled with tomato red fluorescence by lentivirus transduction. After sorting by FACS, fluorescent cells were transduced with an empty lentivirus or encoding an shRNA against PCSK9 (shPCSK9). As can be seen in Figure 6, the cell proliferation as followed by in vivo fluorescence labeling was significantly reduced 24 h and 48 h after injection of the cells transduced with shPCSK9.

## 4. Discussion

Lipid metabolism plays a central role in liver oncogenesis and the important role of PCSK9 in lipid homeostasis places it at the center of the stage as an attractive target in liver cancers [1]. PCSK9 modulation of lipid metabolism arises not only from LDLR binding and targeting for lysosomal destruction, but also from the degradation of other lipoprotein receptors such as VLDLR, ApoER2, CD36 and LRP1. Therefore, it was not inconceivable to expect the alteration of lipid homeostasis we have observed in liver cancer cells in the absence of PCSK9; lipid receptors madly play when PCSK9 is away. 

We report in this paper many anti-oncogenic features of anti-PCSK9 approaches, including the reduction in cell proliferation and the triggering of lipid cytotoxicity in liver tumor cells. Lipid metabolism is strongly affected by this approach, by which disruption probably leads to a massive accumulation in intracellular lipid and higher peroxidation of fatty acids, probably reflecting some metabolic exhaustion leading to overwhelming oxidative stress. The increased oxidative burden and failure of protective measures trigger irreversible cell damage and death by ferroptosis.

The ferroptotic mode of cell death was discovered in recent years (Figure 5A). It is linked to iron-dependent lipid peroxidation triggering the death process [15]. Unlike other modes of cell death, the distinctive morphological features of ferroptosis are obvious at the mitochondrial level with shrinkage of the organite, increased membrane density and the reduction or disappearance of mitochondrial cristae. The integrity of the cell membrane is not affected and the nucleus is normal in size with no chromatin condensation [15,16,17]. 

Herein, we recognize all these ferroptotic morphological features in cells silenced for PCSK9. Moreover, our search for a potential ferroptotic signaling pathway singled out the Keap1/Nrf2 axis. Indeed, PCSK9 depletion led to a significant reduction in Nrf2, which is the chief coordinator of the antioxidant response machinery [18]. Under normal conditions, Keap1 controls the basal cytoplasmic level of Nrf2 by promoting its degradation by the ubiquitin–proteasome system. Under oxidative stress conditions, Keap1, as a redox sensor, gets oxidized and dissociates from Nrf2, which becomes activated and translocates into the nucleus, where it can induce the transcription of different branches of the antioxidant defense system (Figure 5A). In our experimental context, Nrf2 was downregulated in the absence of PCSK9 and failed to launch effective antioxidative responses, as seen by the lack of activation of FTH1, one of the Nrf2 downstream targets.

It is unclear whether PCSK9 nuclear localization, which we reported here, has any direct connection with Nrf2 regulation and activity. The hypothesis of a possible causative correlation may be attractive, but the question deserves further exploration. 

Of note, Nrf2 was reported to be hyperactivated in HCC, promoting the survival of cancer cells and also conferring cellular resistance to chemotherapeutic drugs [19,20,21]. Interestingly, among the main mechanisms accounting for the biological effects of sorafenib, the only approved first-line drug for advanced HCC is the induction of ferroptosis [22], probably by inhibiting the SLC7A11 transporter [15]. Although sorafenib treatment improves survival to some extent, unfortunate severe adverse effects and emerging resistance make it an unsatisfactory therapeutic approach [23]. Interestingly, many strategies to improve sorafenib resistance are aimed at modulating ferroptosis, which is thus emerging as a potential new weapon in the fight against cancer. Some of these strategies are targeting Nrf2 itself. Indeed, the genetic or pharmacological inhibition of Nrf2 in HCC cells makes them more sensitive to the action of sorafenib and even overcomes chemoresistance through the induction of ferroptosis [24]. 

Above all, since the disruption of PCSK9 inhibits the anti-ferroptosis p62-Keap1-Nrf2 pathway, one could speculate that a combination therapy of anti-PCSK9 with sorafenib would alleviate drug resistance and improve prognosis.

In parallel to its involvement in ferroptosis through the p62-Keap1-Nrf2 pathway, PCSK9 may have an impact on this death process through a mechanism that depends on its cholesterol-regulating functions. Indeed, by enhancing lipid/lipoprotein uptake, PCSK9 deficiency may enrich the membrane using PUFAs, which are sensitive to lipid peroxidation and are one of the essential elements for ferroptosis [25]. Free PUFAs can be esterified into membrane phospholipids before being oxidized and thereby inducing ferroptotic signals. Moreover, among some of the highly produced/imported phospholipids following PCSK9 depletion is phosphatidylethanolamine (PE), which was increased 2 to 4 times (Figure 4). Oxidized PEs are the key phospholipids acting as death signals to induce ferroptosis in cells [26]. In this process, 15-lipoxygenase is an important contributor to pro-ferroptosis PE peroxidation, capable of generating doubly- and triply-oxygenated diacylated PE species. Tocopherols and vitamin E suppress this oxygenation and protect against ferroptosis [26]. Therefore, PCSK9 inhibition may trigger ferroptosis in lipid-dependent and -independent fashions. 

Apart from its role in lipid homeostasis, PCSK9 is involved in various signaling pathways including antiviral activity, apoptosis and more recently, anti-tumor immune responses. Indeed, Liu et al. [27] demonstrated that the blockade of PCSK9 can boost the immunotherapeutic efficacy of the anti-programmed death 1 (PD-1) approach. The research involved PCSK9 in the degradation of the major histocompatibility complex class 1 (MHC1), hindering its recycling at the cell surface. Hence, the depletion of this enzyme results in high cellular expression of MHC1, leading to massive infiltration of cytotoxic T cells.

As per our results, PCSK9 may now modulate cancer survival and resistance by an additional mechanism connected to anti-oxidative housekeeping activities. Besides its critical role in modulating lipid metabolism and fluxes, our research brings out a previously unknown function of this intriguing enzyme, that is, maintaining the redox homeostasis via the p62/Keap1/Nrf2 axis. 

The inhibition of PCSK9 led to excessive lipid accumulation and created a void in the defense against oxidative stress, thereby enhancing the vulnerability of cancer cells to ferroptosis. Specific targeting of PCSK9 in hepatic cancer cells showed strong beneficial outcomes and novel mechanistic insights highlighting a new player in cancer cell death by ferroptosis. Taking into account all these anti-tumoral effects of anti-PCSK9 approaches and the existence of a wide variety of therapeutic strategies of the PCSK9 blockade (monoclonal antibodies, small molecule and peptide inhibitors, antisense oligonucleotides, siRNA, etc.), we believe this enzyme is a very valuable and attractive target for the potential treatment of liver cancers. 

## Data Availability

To check the expression level of our gene of interest, two different datasets were selected: Hepatoblastoma—López-Terrada—55—fRMA—u133p2 (GEO ID: gse75271) [11] and Tumor HCC—Wu—134—MAS50 (GEO ID: gse45436) [12]. Expression levels of lipid-related genes in hepatic cell lines were generated from the transcriptomic data collection carried out by our team [13].
